# Oryza Ceramax in Dermatologic Care: A Multi-pathway Approach to Skin Hydration and Barrier Repair

**DOI:** 10.7759/cureus.100886

**Published:** 2026-01-06

**Authors:** Abhishek De, K.N. Sarveswari, Sunil Tolat, Sunaina Hameed, Shilpa Bhat, Sanjay Jain, Onkar C Swami

**Affiliations:** 1 Dermatology, Calcutta National Medical College and Hospital, Kolkata, IND; 2 Dermatology, Raj Kamal Skin Clinic, Chennai, IND; 3 Dermatology, B.J. Government Medical College, Sassoon General Hospital, Pune, IND; 4 Dermatology, Skin.Health Advanced Dermatology Center, Bengaluru, IND; 5 Dermatology, Subodha Skin and Cosmetic Clinic, Bengaluru, IND; 6 Medical Services, Alembic Pharmaceuticals Ltd, Mumbai, IND

**Keywords:** ceramide-cholesterol-fatty acid ratio, dry skin, moisture loss, next-generation moisturizers, oryza ceramax, skin barrier repair, transepidermal water loss (tewl)

## Abstract

Environmental stressors, including climate change, pollution, and lifestyle factors, can disrupt the skin barrier, leading to dryness and exacerbating conditions such as atopic dermatitis (AD), acne, and psoriasis. Effective barrier repair requires maintaining hydration and lipid balance, particularly the ceramide-cholesterol-fatty acid ratio of 3:1:1, which is recommended by dermatological societies for optimal skin restoration. Although traditional moisturizers provide hydration through occlusives, humectants, and emollients, they often do not achieve sustained barrier repair or adequate intracellular hydration. Next-generation moisturizers are designed with bioactive ingredients that aim to both hydrate and support barrier repair by reducing inflammation, modulating the microbiome, and promoting skin homeostasis. Oryza Ceramax (Alaina Healthcare; Alembic Pharmaceuticals Pvt. Ltd., Vadodara, India), a next-generation moisturizer incorporating a 3:1:1 ceramide-cholesterol-fatty acid ratio, includes aquaporin (AQP) boosters, naturally sourced betaine, saffron extract, hyaluronic acid, and other bioactive components that resemble the skin’s natural lipid composition. Clinical evidence supports the efficacy of ceramide-dominant formulations in improving hydration and reducing transepidermal water loss (TEWL), with studies reporting TEWL reductions of approximately 10% and hydration improvement lasting up to 72 hours. Oryza Ceramax is formulated to align with dermatologic recommendations for use in dry or impaired skin and is free from parabens, alcohol, mineral oil, and soap (PAMS-free). Its formulation characteristics are consistent with evidence-based principles for skin barrier protection and hydration maintenance. This narrative review examines the science underlying multi-pathway approaches to skin barrier repair and hydration, using Oryza Ceramax as an example of a ceramide- and AQP-based formulation. The findings highlight emerging strategies in moisturizer design but also emphasize the need for independent, well-controlled clinical studies to validate these observations.

## Introduction and background

Dry skin and skin barrier dysfunction are highly prevalent across populations. Atopic dermatitis (AD) and psoriasis affect approximately 3%-10% and 0.9%-8.5% of adults worldwide, respectively. Dry skin (xerosis cutis) affects over 50% of adults, with a pooled prevalence of 53% (95% CI: 36%-69%), particularly common among older individuals and those in nursing homes or developed countries. The prevalence appears similar between males and females [1,2].

Environmental stressors contribute significantly to this burden. Studies have shown that low humidity and cold/dry weather can impair barrier function, increasing transepidermal water loss (TEWL) and reducing stratum corneum hydration under certain conditions [3]. TEWL is described as an indicator of stratum corneum barrier integrity, measured as the amount of water that passively evaporates from the skin surface [4]. Extreme weather events, including heat waves and fluctuating humidity, can disrupt the skin’s natural barrier and microbiome, increasing vulnerability to inflammation and infection [5,6]. In the Indian context, artificial ventilation systems such as air conditioning reduce ambient humidity and contribute to dehydration of the skin [7]. While urbanization and pollution are recognized risk factors, only air pollution has been consistently associated with dermatologic manifestations such as acne, hyperpigmentation, AD, and psoriasis [8,9].

Traditional moisturizers have long been the cornerstone of managing skin conditions such as AD, psoriasis, and xerosis cutis. However, their effectiveness is limited by the lack of essential components needed for barrier repair, suboptimal pH, absence of anti-inflammatory properties, and inclusion of potential irritants or allergens [10-12]. Peer-reviewed studies support the use of formulations containing urea, ceramides, or anti-inflammatory agents for clinically meaningful relief [11-13].

Despite these advances, current literature remains fragmented, with limited comparative data on moisturizers that address both extracellular and intracellular hydration or target aquaporin (AQP) pathways. Existing reviews often focus on single ingredients rather than integrated, multi-pathway formulations that restore lipid balance, osmotic regulation, and microbial homeostasis. Therefore, the present narrative review aims to critically evaluate multi-pathway hydration and barrier repair strategies, with emphasis on ceramide- and aquaporin-based formulations. Using Oryza Ceramax (Alaina Healthcare; Alembic Pharmaceuticals Pvt. Ltd., Vadodara, India) as an example, this review synthesizes current evidence on ceramide-cholesterol-fatty acid ratio-based and AQP-activating moisturizers, discusses their mechanistic rationale, and identifies key gaps requiring validation through independent, randomized clinical trials.

## Review

Methods

A narrative literature search was conducted in PubMed, Scopus, and Google Scholar databases using combinations of keywords including “skin barrier repair,” “ceramide moisturizer,” “aquaporin-3,” “osmolyte,” “betaine,” “hyaluronic acid,” “saffron extract,” and “Oryza Ceramax.” Publications between 2000 and 2025 were reviewed, including clinical trials, mechanistic studies, and dermatologic guidelines. Reference lists of included articles were screened for additional sources. Evidence from both peer-reviewed and regulatory consensus documents (e.g., the European Task Force on Atopic Dermatitis (ETFAD), the American Academy of Dermatology Association (AAD)) was also considered to describe mechanistic, clinical, and formulation-related insights.

Pathophysiology of Skin Barrier Dysfunction

The skin barrier serves as the body’s primary defense against external insults and internal dysregulation. It consists of a complex network of lipids, proteins, and corneocytes that together maintain hydration and protect against pathogens and irritants [14]. Disruption of this barrier leads to increased TEWL, inflammation, and microbial imbalance, hallmarks of conditions such as AD, psoriasis, and acne [15] .

**Figure 1:**
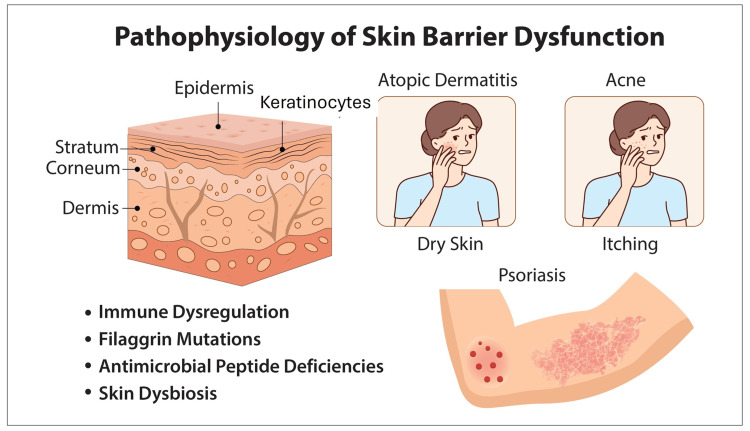
Underlying mechanism resulting in various skin disorders. Illustration of skin barrier dysfunction showing affected skin layers, associated immune and microbial factors, and resulting conditions like atopic dermatitis, acne, and psoriasis. Image credits: Authors.

Recent studies emphasize that filaggrin mutations, altered lipid metabolism, cytokine-driven inflammation, and microbiome dysbiosis play critical roles in barrier dysfunction (Figure 1) [15]. Lipid depletion reduces lamellar body secretion, while cytokines such as interleukin-4 (IL-4) and interleukin-13 (IL-13) inhibit ceramide synthesis and impair lipid organization [16]. These molecular disruptions contribute to dryness, pruritus, and heightened permeability to allergens [17].

Understanding these mechanisms provides the rationale for barrier-repair therapies that restore lipid homeostasis and normalize epidermal hydration through ceramide replenishment, osmolyte supplementation, and aquaporin activation. Such multi-targeted approaches are increasingly reflected in emerging moisturizer designs aimed at restoring both structural and functional integrity of the stratum corneum [10].

Role of Moisturization

Moisturization is a central component of dermatologic therapy and daily skin care. A healthy stratum corneum depends on an intact lipid barrier and adequate water content, both of which maintain elasticity, microbial balance, and immune regulation [10]. Disruption of this equilibrium through intrinsic (genetic, inflammatory) or extrinsic (environmental, occupational) factors leads to TEWL and barrier dysfunction [8,10,13,15,17].

The American Academy of Dermatology recommends routine moisturization to maintain barrier integrity and prevent flares in chronic dermatoses such as AD and psoriasis [10]. Moisturizers generally consist of three major classes of ingredients, occlusives, humectants, and emollients, that collectively reduce water loss, increase surface hydration, and improve tactile smoothness. Occlusives (e.g., petrolatum, dimethicone) form a hydrophobic film to minimize TEWL; humectants (e.g., glycerol, urea) attract water from deeper layers; and emollients (e.g., esters, triglycerides) fill intercellular gaps to soften the skin [10].

Despite their ubiquity, traditional moisturizers provide only superficial or short-term hydration. Clinical evidence indicates that formulations lacking physiological lipids achieve transient hydration lasting four to eight hours, whereas ceramide-containing formulations maintain hydration and TEWL reduction for up to 24-48 hours [13,18]. In elderly subjects with xerosis, Lueangarun et al. (2019) demonstrated that a ceramide-containing moisturizer improved skin hydration by 26% and reduced TEWL by 15% compared with a hydrophilic cream over 28 days [13]. Similarly, Spada et al. (2021) reported a statistically significant TEWL reduction (p < 0.05) and sustained hydration improvement over four weeks using a ceramide-dominant cream and cleanser regimen [18].

These findings underscore the clinical importance of barrier-targeted moisturizers. Traditional over-the-counter products often lack the optimal lipid ratios, humectant balance, and pH required for sustained barrier repair. Some formulations may contain irritant preservatives or surfactants, leading to contact sensitization or reduced adherence, particularly among individuals with sensitive skin [10,12].

In addition to formulation quality, adherence and accessibility are critical determinants of therapeutic success. Moisturizers that are cosmetically acceptable, affordable, and easy to apply are more likely to be used consistently. Studies indicate reduced acceptance when moisturizers are greasy, cause stinging, or have unpleasant odor [12,19]. Thus, the effectiveness of any moisturizer is closely linked not only to its composition but also to user experience and socioeconomic considerations [19].

Gap in the Current Moisturizer Offerings

Although moisturizers remain the mainstay for managing xerosis and inflammatory dermatoses, many products do not fully address the multifactorial nature of skin barrier dysfunction. Conventional formulations often rely on synthetic lipids or occlusive agents that restore surface hydration but fail to replenish intracellular water or natural moisturizing factors. Recent research has identified that effective skin repair requires a layer-by-layer hydration approach, restoring both extracellular lipids and intracellular osmotic balance [10,11]. Formulations that focus solely on occlusion or superficial emollience are insufficient for long-term outcomes. For instance, even well-established emollient bases without ceramide supplementation show limited improvement in TEWL and fail to restore natural lipid synthesis pathways [11,13].

Furthermore, moisturizers are rarely optimized for different skin types, climatic conditions, or anatomical sites, which can influence efficacy and patient satisfaction. Economic accessibility also remains a concern, as many advanced formulations are priced higher, limiting adherence and real-world benefit [8,19].

From a clinical and research perspective, three key gaps persist: 1. Lack of integration between molecular and clinical endpoints, few studies correlate biochemical mechanisms (lipid synthesis, AQP activation) with clinical measures like TEWL or Eczema Area and Severity Index (EASI) improvement. 2. Limited transparency of evidence, most available data are derived from small, industry-sponsored or in vitro studies rather than independent, randomized clinical trials. 3. Need for standardized evaluation criteria, guidelines exist for ceramide ratios and pH optimization, but there is no universal framework for comparing next-generation moisturizer efficacy.

Therefore, there remains an unmet need for affordable, evidence-based, multi-pathway formulations that simultaneously address lipid replenishment, osmolyte balance, and cellular hydration. This research gap provides the rationale for reviewing ceramide- and AQP-based technologies, as exemplified by formulations such as Oryza Ceramax, which integrate these mechanisms into a single barrier-repair strategy.

An Era of New-Generation Moisturizers

Emerging evidence highlights that each functional element of the epidermal barrier, lipids, aquaporins, osmolytes, and tight junctions, represents a potential therapeutic target for improving skin hydration and resilience. Next-generation moisturizers are therefore designed not merely to occlude water loss but to deliver bioactive components that address the underlying mechanisms of barrier dysfunction [10,20]. Among these targets, aquaporins, particularly AQP3, play a pivotal role in regulating water and glycerol transport across keratinocyte membranes [21,22]. Studies have shown that enhancing AQP3 expression can improve hydration and accelerate wound healing [22]. Several commercially available moisturizers now include ingredients such as glyceryl glucoside, an AQP3 inducer demonstrated to increase AQP3 expression in vitro and ex vivo [23]. These findings suggest that products incorporating AQP-activating compounds may facilitate not only hydration but also repair of the stratum corneum.

In parallel, organic osmolytes, such as betaine and taurine, have emerged as complementary agents supporting barrier function by stabilizing intracellular osmotic balance and strengthening tight junctions [24]. Their contribution extends beyond hydration; by upregulating claudin and occludin proteins, they enhance skin barrier integrity and reduce TEWL [24]. Moisturizers combining ceramides, osmolytes, and AQP boosters thus represent a multi-target approach to restoring barrier function, a strategy shared by various ceramide-dominant or osmoprotective formulations available in the clinical setting (e.g., EpiCeram®, CeraVe®, or physiologic lipid creams) [17,22,24-26]. Collectively, this evidence positions next-generation moisturizers as vehicles for biofunctional restoration of the epidermal barrier rather than simple hydration. Continued comparative research is essential to determine whether these formulations achieve clinically superior outcomes versus conventional products.

Need for Parabens, Alcohol, Mineral Oil, and Soap (PAMS)-Free Moisturizers

Dermatologic societies increasingly emphasize minimizing exposure to potentially allergenic excipients in daily skin care. The American Academy of Dermatology Association (AAD, 2017) recommends using moisturizers free from parabens, alcohol, mineral oil, and soap, collectively referred to as parabens, alcohol, mineral oil, and soap (PAMS)-free, particularly for patients with sensitive or compromised skin [19]. Clinical and consumer analyses have demonstrated that products labeled “free from fragrance or preservatives” are associated with lower rates of irritant or allergic contact dermatitis, improved adherence, and better patient-reported satisfaction [19,27]. In pediatric and atopic populations, fragrance-free and preservative-minimized formulations correlate with reduced flare frequency and improved quality of life scores [27].

In this context, the PAMS-free concept is not a marketing descriptor but a dermatologic safety parameter for allergen avoidance. Excluding common sensitizers reduces the risk of barrier irritation and supports long-term treatment adherence. Formulations adhering to these standards, such as those aligned with atopic dermatitis (eczema) guidelines, contribute to safer and more tolerable barrier repair therapies [19,28].

Properties of a New-Generation Moisturizer

An effective moisturizer should address hydration, lipid balance, and skin tolerability in accordance with dermatologic guidelines [8,29-31]. The key evidence-based properties include:

Osmohydration and cellular-level hydration: Incorporation of humectants and osmolytes that enhance intracellular water balance and support tight junction integrity [10,22].

Minimization of TEWL: Use of physiological lipid ratios, typically ceramide:cholesterol:fatty acid in a 3:1:1 ratio, to mimic and restore the natural lipid matrix. The European Task Force on Atopic Dermatitis (ETFAD, 2009; 2020) and Korean Atopic Dermatitis Association (2015) specifically recommend such ratios for optimal barrier recovery [29-31].

Barrier restoration and lipid replenishment: Combination of ceramides with cholesterol and free fatty acids facilitates lipid synthesis in granular keratinocytes, supporting long-term repair [20,27].

Optimal pH (~5.5): Maintaining slightly acidic pH enhances β-glucocerebrosidase and acid sphingomyelinase activity, enzymes critical for lipid processing, and promotes a balanced microbiome.

Dermatologic safety: Non-comedogenic, hypoallergenic, and PAMS-free characteristics minimize irritant potential, especially in AD and xerosis. These principles are endorsed by AAD and ETFAD recommendations [19,28,31].

Cosmetic acceptability and affordability: Lightweight, fast-absorbing formulations improve patient compliance; accessibility supports consistent, long-term use [32].

By integrating these parameters, new-generation moisturizers aim to achieve multi-dimensional skin barrier restoration through evidence-based design rather than purely cosmetic optimization.

Introducing Oryza Ceramax: Biomimetic Hydration and Barrier Restoration

Oryza Ceramax is a ceramide-dominant, bioactive-based formulation developed to restore epidermal barrier function through multi-pathway hydration mechanisms. Its composition reflects the 3:1:1 ratio of ceramide, cholesterol, and fatty acids, consistent with ETFAD and other dermatologic recommendations for optimal lipid replenishment [29-31]. In addition to lipid replacement, the formulation integrates hyaluronic acid (HA), betaine, saffron extract, and AQP-activating bioactives that support osmotic regulation and hydration. The combination of humectant (HA), osmolyte (betaine), and barrier lipid elements provides a layered approach to hydration, targeting both extracellular and intracellular compartments.

Evidence regarding Oryza Ceramax’s components is derived from multiple data streams. In vitro and ex vivo studies have shown that ceramide-rich formulations can improve lipid lamellae organization and reduce TEWL by 10%-20% after two weeks of application [11,12,17]. Clinical data on ceramide-dominant moisturizers demonstrate improved hydration and skin smoothness in patients with AD and xerosis compared with standard emollients [13,18,33].

Role of AQP in Moisturization

**Figure 2:**
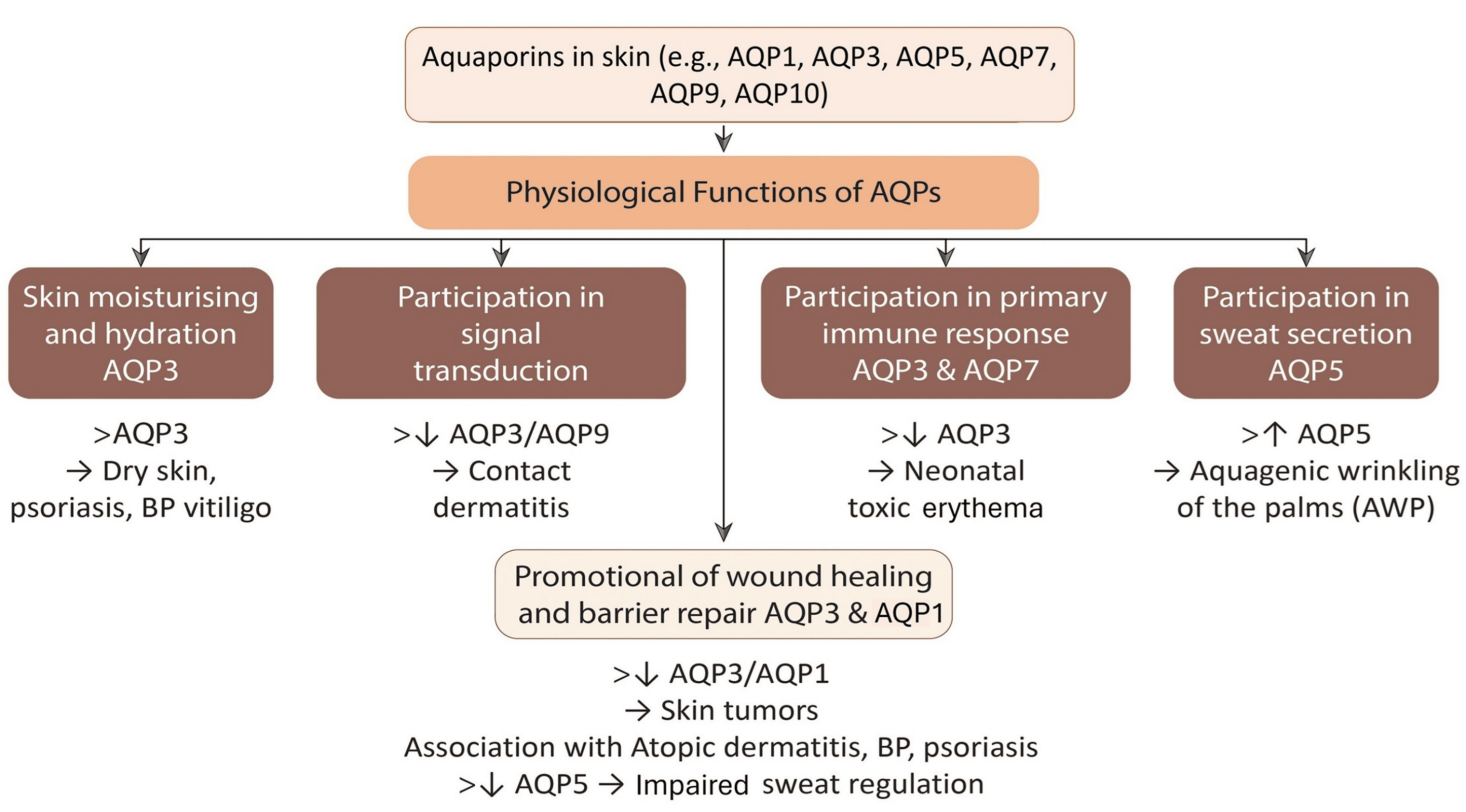
Mechanism of action of aquaporins. AQPs in the skin play key roles in hydration, immune response, signal transduction, and sweat secretion. Dysregulation of specific AQPs is associated with conditions such as psoriasis, contact dermatitis, and impaired barrier repair. AQPs: aquaporins; BP: bullous pemphigoid. Image credits: Authors (adapted from [35] under Creative Commons CC-BY 4.0).

AQPs are integral membrane proteins that facilitate the transport of water, glycerol, and small solutes across epidermal layers (Figure 2). AQP3, predominantly expressed in keratinocytes, is a key regulator of epidermal hydration and elasticity [22]. Experimental studies indicate that AQP3 knockout mice exhibit delayed barrier recovery, increased TEWL, and reduced stratum corneum hydration, underscoring the importance of this channel in skin physiology [23]. AQP3 in the skin is regulated by interconnected pathways: Epidermal growth factor receptor-mitogen-activated protein kinase pathway/phosphatidylinositol 3-kinase (EGFR-MAPK/PI3K) signaling upregulates its expression, while retinoic acid enhances AQP3 and nicotinamide blocks this effect. AQP3 colocalizes with phospholipase D2 (PLD2) in caveolin-rich domains, where its glycerol transport enables production of phosphatidylglycerol, linking AQP3 to lipid-driven keratinocyte differentiation. Its levels also follow circadian rhythms, respond to peroxisome proliferator-activated receptor gamma (PPARγ) activation, and modulate oxidative signaling by transporting H₂O₂ [22,23,34]. The key AQPs involved in skin hydration include AQP1, AQP3, AQP5, and AQP9, each contributing to different aspects of skin barrier function (Figure 2). These mechanisms integrate to coordinate keratinocyte proliferation, differentiation, hydration, migration, and stress responses, making AQP3 a central molecular hub in skin physiology and disease.

Benefits of AQP Boosters in Moisturizers

AQP-activating compounds such as glycerol and glyceryl glucoside have demonstrated improved hydration and TEWL reduction in both ex vivo human skin and controlled clinical settings [23]. For example, Hara-Chikuma et al. (2015) observed a 16% increase in hydration and a 12% TEWL reduction following topical AQP3 activation [34]. Similar AQP-boosting approaches are utilized in formulations containing glyceryl glucoside (e.g., Eucerin®) and betaine-enriched moisturizers [4,23,36-39]. Within this scientific framework, Oryza Ceramax is an example of a formulation incorporating AQP-supportive and osmoprotective components to promote sustained hydration.

Role of Betaine in Hydration and Its Benefits

Betaine, also known as trimethylglycine, is an organic osmolyte recognized for its hygroscopic properties, making it a valuable ingredient in advanced skincare formulations. It plays a crucial role in maintaining cellular hydration by regulating water balance and protecting skin cells from environmental stressors such as UV radiation and dehydration [24]. As a natural osmolyte, betaine helps preserve cell volume and osmotic balance under stress conditions, while stabilizing proteins and cell membranes and reducing oxidative stress and inflammation [24].

Tight junction enhancement and barrier function: Recent studies have expanded the understanding of betaine’s impact on skin barrier function, particularly through its effects on tight junctions (TJs). Betaine enhances the expression of TJ proteins such as claudin-1, claudin-4, and occludin, which are essential for maintaining the skin’s water barrier and preventing TEWL [24].

Cell volume regulation and osmotic balance: Betaine’s role as an osmolyte extends to regulating osmotic pressure within skin cells, particularly under environmental stress [7]. It accumulates in keratinocytes to prevent cellular shrinkage and maintain cell volume, which is critical for preserving skin elasticity and hydration, especially in dry or damaged skin. This osmoprotective function complements aquaporin-3 (AQP3)-mediated water transport, contributing to intracellular hydration and improved skin resilience [24].

Additional barrier protection via transporter activation: Beyond its humectant properties, betaine upregulates its transporter, betaine-GABA transporter-1 (BGT-1), which is expressed throughout the epidermis, especially in the granular layer [15]. BGT-1 facilitates betaine uptake into skin cells, enabling its protective effects. This mechanism enhances the skin’s ability to respond to environmental aggressors by fortifying the epidermal barrier [24].

Clinical and in vitro evidence: In vitro studies using human keratinocytes demonstrated that betaine significantly increased transepithelial electrical resistance (TEER), a marker of TJ function, at 48 hours (p < 0.001), with sustained effects at 72 hours (p < 0.001). Even when added to already developed TJs, betaine further enhanced TEER (day 6, p = 0.0022), indicating its ability to reinforce the skin’s moisture retention capacity [24]. Clinical data, though limited, suggest that moisturizers containing betaine improve subjective dryness scores and maintain hydration for up to 24 hours post-application [4,38]. These findings point to potential synergistic benefits when betaine is combined with ceramides and AQP-based actives, reinforcing both osmotic and lipid-dependent hydration pathways.

Benefits of Betaine in Moisturizers

Incorporating betaine into moisturizers offers multiple skin benefits:

Improved hydration: Acts as a humectant, drawing and retaining water within the skin.

Strengthened skin barrier: Enhances TJ protein stability and osmotic regulation, reducing TEWL.

Protection against environmental stressors: Maintains cell volume and barrier integrity under UV and dehydration stress.

**Figure 3:**
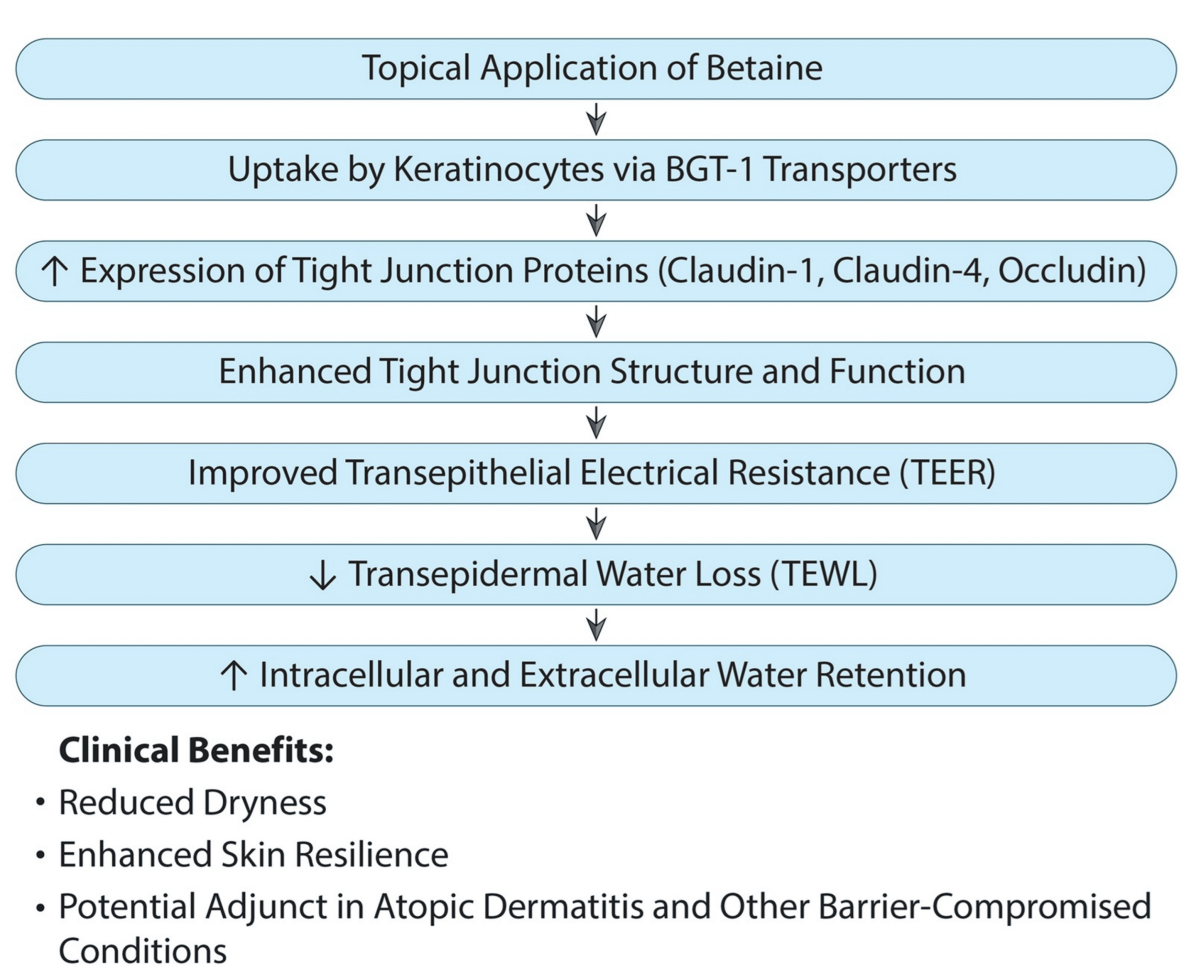
Betaine’s role in skin hydration. Betaine enhances skin hydration by strengthening tight junctions, improving TEER, and reducing TEWL. This leads to better water retention and clinical benefits such as reduced dryness and improved skin resilience. TEER: transepithelial electrical resistance; TEWL: transepidermal water loss; BGT-1: betaine-GABA transporter 1. Image credits: Authors (adapted from [24] under Creative Commons CC-BY-NC).

Thus, betaine represents a mechanistically validated component in next-generation formulations, functioning alongside ceramides and AQP activators to reinforce both osmotic and lipid-dependent hydration pathways (Figure 3).

Natural betaine avoids allergenic concerns associated with synthetic cocamidopropyl betaine: Cocamidopropyl betaine is an amphoteric synthetic detergent widely used in personal care products such as shampoos, cleansers, soaps, and hygiene products due to its mild, non-irritating nature compared to other surfactants such as sodium lauryl sulfate. Its use has significantly increased; in the USA, personal care products using cocamidopropyl betaine increased from 3.3% in 1989 to 6.2% in 1994 [40]. However, it is more likely to cause allergic sensitization, commonly presenting as dermatitis on the eyelids, face, scalp, or neck due to frequent exposure. As its contact sensitization rates continued to rise, it was named the American Contact Dermatitis Society’s (ACDS) Allergen of the Year in 2004 [27,41].

Role of Saffron Extract in Skin Health

Saffron extract (*Crocus sativus* L.) plays a multifaceted role in dermatology, promoting skin health through its antioxidant, anti-inflammatory, and regenerative properties. Its key bioactive compounds, crocin, crocetin, safranal, and picrocrocin, exhibit strong antioxidant activity by reducing reactive oxygen species (ROS), thereby protecting the skin from photoaging and oxidative stress induced by UV radiation [42-44]. These compounds also demonstrate anti-inflammatory effects by inhibiting cytokines such as interleukin-6 (IL-6) and tumor necrosis factor-α (TNF-α), which contribute to skin inflammation and aging​​ [42-44]. Moreover, saffron enhances collagen and hyaluronic acid (HA) synthesis in human dermal fibroblasts and has been shown to significantly increase collagen density in UVB-exposed rat skin, which supports its anti-wrinkle and skin-firming effects​​ [42,43]. It also acts as a natural skin lightening agent through tyrosinase inhibition, making it useful in treating hyperpigmentation disorders such as melasma​​ [42,45]. Additionally, saffron compounds such as picrocrocin and crocetin stimulate dermal fibroblast migration, aiding wound healing and skin regeneration [42]. These properties collectively highlight saffron’s significant potential in cosmetic dermatology.

Role of Hyaluronic Acid in Skin Health

HA plays a vital role in maintaining skin health through its ability to retain moisture, enhance elasticity, and support tissue repair. It is naturally present in the skin's extracellular matrix and binds water to keep the skin hydrated and supple. In aging skin, HA levels decline, leading to dryness, wrinkles, and loss of firmness. Topical, injectable, and oral HA formulations have shown significant benefits in improving skin hydration, reducing wrinkles, and promoting regeneration [46,47]. Additionally, HA-based compounds aid wound healing by modulating inflammation, supporting cell proliferation, and enhancing collagen synthesis [46,48,49].

Comparative Evidence Summary

To contextualize the role of Oryza Ceramax within next-generation moisturizers, a comparative synthesis of ceramide-, AQP-based and similar formulations is provided below. Table 1 integrates primary formulation, interventions, clinical relevance, and outcomes drawn from published peer-reviewed studies.

**Table 1:** Clinical studies evaluating the moisturizers in skin barrier repair. Oryza Ceramax (Alaina Healthcare; Alembic Pharmaceuticals Pvt. Ltd., Vadodara, India), EpiCeram® (PuraCap Pharmaceutical LLC; Primus Pharmaceuticals, Arizona, USA), CeraVe® (L’Oréal India Pvt. Ltd.; L’Oréal, Mumbai, India), and Eucerin® (Beiersdorf AG, Hamburg, Germany); TEWL: transepidermal water loss; EASI: Eczema Area and Severity Index; DLQI: Dermatology Life Quality Index; SCORAD: SCORing Atopic Dermatitis; A/BPO: adapalene/benzoyl peroxide; AEs: adverse events; HECSI: Hand Eczema Severity Index; OR: odds ratio; CI: confidence interval; RCT: randomized controlled trial; FA: fatty acid; HA: hyaluronic acid; AQP3: aquaporin-3; TSLP: thymic stromal lymphopoietin; TARC: thymus and activation-regulated chemokine; AD: atopic dermatitis; NMF: natural moisturizing factor; pH: potential of hydrogen.

Formulation/Study Type	Study Design	Author and Year	Intervention	Outcome Measures	Results
EpiCeram® (3:1:1 ceramide:cholesterol:FA)	Phase I open-label clinical trial	Lowe et al., 2012 [33]	Daily ceramide-dominant triple-lipid cream in neonates	Safety, compliance, eczema incidence, TEWL, hydration, pH	High compliance (80% used ≥80% of time); no AEs; lower TEWL suggests preventive barrier benefit
EpiCeram®/triple lipid therapy	Narrative review summarizing RCTs and clinical trials	Elias, 2022 [26]	Physiologic lipid therapy (3:1:1 ceramides:cholesterol:FAs)	SCORAD, pruritus, sleep, barrier integrity	Triple lipid therapy restores barrier, reduces inflammation, improves SCORAD/pruritus, comparable to mid-potency steroids
CeraVe® (ceramides + HA) vs. hydrophilic cream	Split-site, double-blind RCT	Lueangarun et al., 2019 [13]	Ceramide 1/3/6-II moisturizer vs. hydrophilic cream in elderly xerosis	Hydration, TEWL, skin pH, wrinkles, texture	Ceramide group: significantly improved hydration, reduced TEWL/pH, better wrinkle/texture outcomes up to 7 days
Ceramide-dominant cream + cleanser	Randomized, double-blind, placebo-controlled trial (28 days)	Spada et al., 2021 [18]	Ceramide-dominant cream/cleanser vs. placebo	EASI, TEWL, hydration, DLQI, satisfaction	TEWL and hydration significantly improved vs. placebo; EASI improved in both groups; higher satisfaction in ceramide arm
CeraVe® cleanser and lotion in acne treatment	Double-blind, randomized, controlled trial	Draelos et al., 2023 [50]	Ceramide foaming cleanser + moisturizer with A/BPO (adapalene (0.3%) and benzoyl peroxide (2.5%))	TEWL, dryness, erythema, scaling, acne lesions, satisfaction	Ceramide group: significantly lower TEWL, dryness, erythema; no interference with acne therapy; 97.8% satisfaction
Ceramide cream for hand dermatitis	Double-center, randomized trial	Filon et al., 2023 [51]	Ceramide-containing cream + training vs. non-ceramide	HECSI, TEWL, clinical improvement	Ceramide group: better HECSI and TEWL; 63% improved vs. 50% control
Eucerin® Aquaporin active	Ex vivo mechanistic + small clinical study	Schrader et al., 2012 [23]	Glyceryl glucoside (AQP3 upregulator)	AQP3 expression, TEWL, hydration	Increased AQP3 expression; +16% hydration; −12% TEWL in small clinical dataset
Physiologic lipid creams (ceramides + cholesterol)	Observational/comparative studies	Spada et al., 2021 [18]	Ceramide-dominant barrier therapy	TEWL, hydration	TEWL reduction 10%-15% over 14 days; improves lipid matrix and barrier integrity
Multi-ingredient moisturizer (glycerin + HA + betaine + minerals)	Multicenter, 8-week clinical study in AD	Fukushima et al., 2014 [4]	Lotion/emulsion/cream containing glycerin, hyaluronic acid, betaine, and seawater minerals	Skin symptoms, TEWL, high-frequency conductance, stratum corneum TSLP and TARC	Significant reduction in TEWL, erythema, dryness, itching; increased hydration; significant reductions in TSLP and TARC, indicating improved barrier physiology and inflammation control; no adverse events
Moisturizer after hand hygiene	Clinical evaluation in healthcare workers	Filatov et al., 2024 [36]	Moisturizing cream applied after repeated hand hygiene	TEWL, hydration, dryness, irritation	Improved hydration, reduced TEWL, mitigated dryness/irritation following frequent washing; supports moisturizers for occupational barrier recovery
Betaine and NMF containing moisturizer in xerosis	Double-blind, vehicle-controlled study	Weber et al., 2012 [37]	Barrier-repair moisturizers (rich and light) in xerosis	TEWL, lipid lamellae integrity, barrier recovery	Significant TEWL reduction and improved barrier recovery; decreased visible dryness
Low molecular weight HA topical gel for seborrheic dermatitis	Prospective, observational, non-blinded safety and efficacy study	Schlesinger et al., 2014 [47]	Hyaluronic acid sodium salt gel (0.2%) with Cetaphil Gentle Cleanser	Erythema, pruritus, and provider global assessment on 5-point scale	Scale, erythema, pruritus evaluation results remained same or continued to improve, no adverse event, excellent tolerability
Oryza Ceramax (3:1:1 lipids + HA, betaine, saffron)	Preliminary data	Unpublished data: Single blind comparative study. Evaluation & Comparison of Efficacy and Safety of Skin Care Formulations Versus Untreated Control. September 12, 2024	Multi-pathway approach: lipid replenishment + AQP activation + osmotic regulation	Lipid organization, hydration markers	In vitro and early-stage data indicate improved lipid organization and hydration

Guideline Recommendations

Guideline and recommendations for moisturizer use in skin issues including AD mention the use of ceramide-based moisturizers (Table 2).

**Table 2:** Guideline recommendations for moisturizer use in AD. AD: atopic dermatitis; EADV: European Academy of Dermatology and Venereology; SC: stratum corneum.

Organization/Guideline	Recommendation	Key Notes
Korean Atopic Dermatitis Association (KADA), 2015 and 2025 [15,52]	Recommends emollients with lipid ratios mimicking physiological composition (e.g., 3:1:1 or 1:1:1)	Lipid-balanced formulations (ceramides:cholesterol:fatty acids) support barrier repair
Asian-Pacific Consensus Guidelines, 2017 [12]	Advocates regular use of moisturizers as maintenance and adjunctive therapy for AD	Supports continuous application to maintain remission
European Task Force on Atopic Dermatitis (ETFAD, 2009) [30]	Recommends 3:1:1 ceramide-dominant formulations for barrier restoration	Focusing on physiological lipid replenishment
ETFAD/EADV Eczema Task Force (2020) [31]	Highlights increased TEWL due to deficiency/imbalance of SC lipids and filaggrin mutations	Emphasizes importance of lipid-replenishing moisturizers
General Dermatology Recommendations [27,53]	Suggests moisturizers be free from mineral oil, soap, and fragrance	Ensuring suitability for sensitive or compromised skin

Benefits of New-Generation Moisturizer (Oryza Ceramax): Combining AQP Boosters; Betaine; Ceramides, Cholesterol, and Fatty Acids in 3:1:1 Ratio; HA and Saffron Extract

Water transport regulation and improved cellular-level hydration: AQPs, particularly AQP3, play a crucial role in regulating water and glycerol transport in the skin, contributing to deep hydration and elasticity. Moisturizers targeting AQP3 enhance skin hydration and improve barrier function​, unlike common moisturizers widely available [22]. AQP boosters increase hydration within cells.

Osmolyte protection: Betaine maintains cell volume under stress conditions such as UV radiation, protecting skin cells from dehydration. Its ability to regulate osmotic balance makes it particularly effective at retaining skin moisture.

Tight junction enhancement: Betaine strengthens the skin barrier by increasing the expression of tight junction proteins such as claudin-1 and occludin, which are essential for preventing water loss [24].

Barrier reinforcement: Betaine enhances structural integrity by supporting intracellular and extracellular water regulation [24]. Saffron extract also improves moisturization [45].

Barrier restoration: The 3:1:1 molar ratio of ceramides, cholesterol, and fatty acids closely mirrors the natural lipid composition of the skin. This combination, along with HA, helps restore the lipid barrier, effectively reducing TEWL and preventing moisture evaporation​ [49]. Ceramides are key to epidermal barrier integrity, whereas cholesterol and fatty acids support lipid synthesis, enhancing the skin's ability to retain moisture. Together, they promote long-term barrier repair, essential for treating conditions such as AD, eczema, psoriasis, dermatitis, and xerosis [29,37].

Wound healing: AQP3 promotes keratinocyte migration and proliferation, aiding wound healing and skin regeneration. This makes AQP-enhanced moisturizers beneficial for skin repair [22,24].

pH 5.5: A pH of 5.5 optimizes β-glucocerebrosidase activity for skin health, enhances antimicrobial defense by inhibiting harmful microorganisms while supporting beneficial microflora, and facilitates the desquamation process by promoting the proteolytic degradation of corneodesmosomes [54].

Thus, the new-generation moisturizer (Oryza Ceramax) offers advanced hydration and barrier restoration by combining AQP boosters, ceramides, cholesterol, fatty acids, naturally sourced betaine, HA, and saffron extract. This unique formulation enhances cellular hydration, strengthens the skin barrier, and supports wound healing, making it ideal for managing dry skin and chronic conditions such as eczema and psoriasis. Clinically shown to deliver noticeable hydration within 15 minutes and maintain moisture for up to 72 hours, Oryza Ceramax also restores the skin barrier within three days of use (Unpublished data: Single blind comparative study. Evaluation & Comparison of Efficacy and Safety of Skin Care Formulations Versus Untreated Control. September 12, 2024). It reduces TEWL by 10%, reinforcing barrier integrity and enhancing skin resilience.

These ingredients may have formulation limitations, including instability of natural actives (e.g., saffron polyphenols), variable penetration of large molecules like HA, and the need for optimized delivery systems to ensure bioavailability and consistent clinical effects [10]. Additionally, botanical and osmolyte components such as betaine or saffron extracts may introduce allergenicity or batch-to-batch variability, requiring careful standardization and safety assessment [44].

## Conclusions

Maintaining an effective skin barrier is essential for skin health as it defends against environmental aggressors, prevents moisture loss, and mitigates the effects of various dermatological conditions. Although offering temporary relief, traditional moisturizers often fail to provide long-lasting hydration or address the root causes of skin barrier dysfunction. The development of next-generation moisturizers such as Oryza Ceramax represents a significant advancement in dermatological care. These formulations incorporate AQP boosters, naturally sourced betaine, and a 3:1:1 lipid ratio of ceramides, cholesterol, and fatty acids, along with HA and saffron extract, delivering comprehensive hydration at both superficial and cellular levels.

Early data suggest potential benefits for sustained hydration and barrier recovery, but these findings remain preliminary. Independent, large-scale clinical studies are needed to validate their efficacy and safety, elucidate molecular mechanisms in vivo, and establish standardized criteria for comparing emerging formulations. By addressing both immediate and long-term hydration needs, next-generation moisturizers offer a holistic approach to skincare, making them an essential tool in the management and prevention of dry skin and barrier-related disorders. Their ability to combine advanced hydration with barrier repair establishes a new paradigm in skincare, ensuring healthier, more resilient skin for a wide range of patients.
